# Erythropoiesis-Stimulating Agents (ESAs) in Chronic Kidney Disease and Cancer-Related Anemia: A Narrative Review of Literature

**DOI:** 10.7759/cureus.93584

**Published:** 2025-09-30

**Authors:** Mounica A Pothacamuri, Adwaith Venugopal, Neha Chandrashekar, Waldyr M Banderas Echeverry, John G Appiah, Saeed AlSalami, Khaled Ahmed, Saya Alasaadi, Abdulaziz Zayed Almutairi, Eziamaka Mbaekwe, Neeraj Bodapati, Ramsha Ali

**Affiliations:** 1 Internal Medicine, Trinity West Medical Center, Steubenville, USA; 2 Medicine, University of Central Lancashire, Preston, GBR; 3 Medicine, JSS Medical College and Hospital, Mysuru, IND; 4 Medicine, Universidad Libre, Cali, COL; 5 Medicine, University of Science and Technology, Accra, GHA; 6 Medicine, Royal College of Surgeons, Dublin, IRL; 7 Medicine, Minia University Faculty of Medicine, El-Minya, EGY; 8 Medicine, University College Dublin, Dublin, IRL; 9 Pharmacy, College of Pharmacy, University of Hail, Riyadh, SAU; 10 Oncology, St. George’s University Hospital, London, GBR; 11 Medicine, Alluri Sitaramaraju Academy of Medical Sciences, Eluru, IND; 12 Medicine and Surgery, People's University of Medical and Health Sciences, Nawabshah, PAK

**Keywords:** daprodustat, erythropoiesis-stimulating agents in anemia, erythropoiesis-stimulating agents in ckd, esas and their effectiveness in kidney disease, esas in cancer related anemia, hypoxia-inducible factor prolyl hydroxylase inhibitors (hif-phis), roxadustat, vadadustat

## Abstract

Anemia associated with chronic kidney disease (CKD) and cancer is conventionally managed with packed red blood cell (PRBC) transfusions or erythropoietin-stimulating agents (ESAs) like epoetin alfa; however, transfusions are limited by complications such as alloimmunization and infection risk, which has led to ESAs becoming the preferred standard of care. Additional therapy may include iron supplementation, which potentially causes complications such as iron overload and infection risks in respective patient populations. The introduction of recombinant human erythropoietin (rhEPO) in 1989 improved anemia management but also raised concerns about adverse cardiovascular outcomes in many studies. Current guidelines promote careful ESA use to balance benefits and risks, while alternatives like hypoxia-inducible factor prolyl hydroxylase inhibitors (HIF-PHIs) show promise in reducing such adverse effects. This review explores ESA trends, challenges, and emerging therapies for anemia in CKD and cancer patients and their implications in clinical use.

The literature search for this narrative review was conducted on PubMed in January 2025. The search was restricted to articles published between January 2020 and January 2025, focusing on randomized controlled trials (RCTs), narrative reviews, systematic reviews, and meta-analyses. The initial PubMed search yielded 454 articles, which were subsequently screened according to the inclusion and exclusion criteria, resulting in a final selection of 58 publications that satisfied the eligibility requirements.

Overall, evidence from these studies suggests that ESAs are considerably beneficial in correcting anemia and lowering the need for blood transfusions in adult patients with CKD. However, concerns about adverse cardiovascular outcomes and their effects on optimal hemoglobin targets have indicated the need to shift treatment approaches. These articles have also suggested recent developments, including the advent of HIF-PHIs and sodium-glucose cotransporter-2 (SGLT-2) inhibitors, which have been shown to offer safer and therapeutically promising alternatives in anemia of CKD and cancer-related anemia. Tailored approaches that take patient-specific factors into account are necessary for optimizing outcomes, suggesting that further research is required to evaluate the efficacy and risks of these novel treatments within clinical settings.

The narrative review has summarized the benefits and drawbacks of ESAs as a widely used treatment for anemia of CKD and cancer-related anemia. Studies identified in this review have shown that ESAs are linked to increased risks of adverse cardiovascular events, tumor progression in cancers, and higher mortality rates. The emerging alternative of HIF-PHIs shows promise in mitigating these adverse risks with a similar treatment efficacy to ESAs. However, there is still a lack of long-term safety data on these treatment options, and future research should focus on determining this risk profile as well as potential dosing strategies to potentially guide the use of HIF-PHIs in future clinical practice as a novel therapeutic alternative for anemia of CKD and cancer-related anemia.

## Introduction and background

Chronic kidney disease (CKD) is a major healthcare concern worldwide. About 10-15% of the global population is estimated to have CKD, which can progress to end-stage renal disease (ESRD) and decrease the quality of life (QoL) in patients [1]. Anemia is also a major concern in patients with CKD, where nearly 4.8 million people in the United States are affected [2]. The prevalence of anemia in CKD in the United States ranges from 20% at stage III to 50% at stage V [3]. The prevalence increases as the glomerular filtration rate (GFR) decreases at a rate <30 mL/min/1.72 m². With the progression of CKD, kidneys lose their capacity to generate erythropoietin (EPO), leading to anemia [4]. Other causes of renal anemia include nutritional deficiencies such as iron, folic acid, and vitamin B12; other concomitant chronic inflammatory states like diabetes and cancer; and another major cause being higher circulating levels of uremia-induced inhibitors of EPO [1,5].

Historically, anemia in CKD was managed with blood transfusions and androgens; however, these approaches were associated with significant complications, including frequent blood transfusions causing complications such as iron overload, increased risk for viral infections, and human lymphocyte antigen allosensitization [4]. Given these complications, there has been a need for newer modalities of treatment such as hypoxia-inducible factor prolyl hydroxylase inhibitors (HIF-PHIs) and erythropoiesis-stimulating agents (ESAs) [6]. ESAs act by stimulating red blood cell production through activation of EPO receptors, thereby improving hemoglobin levels. They remain central to the management of anemia in both CKD and cancer patients; however, their use also carries important clinical risks, particularly cardiovascular complications, which underscore the need for careful therapeutic decision-making.

Further investigations stated this was due to an amino acid glycoprotein hormone synthesized and secreted by the kidneys called EPO. Recombinant erythropoietin (rhEPO) is an artificial version of natural EPO, which was approved for use in 1989 by the U.S FDA [7].

The Kidney Disease: Improving Global Outcomes (KDIGO) guidelines (2012) recommend the use of ESA treatment in targeting hemoglobin levels of 10 to 11.5 g/dL, and European Renal Best Practice (ERBP) treatment should target 10 to 11 g/dL ranges for clinical management of anemia [8].

Several studies suggest that ESAs, such as epoetin alfa and darbepoetin alfa, have long played a key role in the treatment of anemia in the above-discussed populations, as the use of these agents has been demonstrated to show notable improvements in hemoglobin levels, QoL, and overall functional status in several studies [9]. However, recent studies have highlighted several concerns about the potential cardiovascular risks associated with ESA therapy, outlining outcomes of increased risk of mortality, escalation of hypertension, myocardial infarction, and stroke [10]. This subsequently prompted a reconsideration of dosing strategies and further exploration of alternative therapies, such as HIF-PHIs like roxadustat and daprodustat, which have shown promising potential in improving anemia without the same cardiovascular risk profile [7]. This review aims to explore current trends in ESA use and address potential obstacles to desirable therapeutic outcomes in clinical practice. Our goal is also to identify areas for further improvement relating to the role of ESAs in anemia management among CKD and cancer patients. We also aim to look closely at the benefits, growing risks, and promising alternatives to conventional therapies.

## Review

Study selection and characteristics

The literature search for this narrative review was conducted on PubMed in January 2025 to identify studies on the use of ESAs in CKD-associated anemia. Search terms included “erythropoiesis-stimulating agents in CKD,” “erythropoiesis-stimulating agents in cancer-related anemia,” and “ESA and their effectiveness in kidney disease.” The search was restricted to articles published in English between January 2020 and January 2025, focusing on randomized controlled trials (RCTs), narrative reviews, systematic reviews, and meta-analyses involving adult populations aged 18-99 years. The inclusion criteria were studies on ESAs in CKD, ESAs in cancer-related anemia, adults aged >18 years, and publications from the last five years. Exclusion criteria comprised non-English studies, pediatric populations, and pregnant women. The initial literature search had yielded 454 publications, and upon completing the entire screening process, which includes applying the above-mentioned criteria, the literature search found 58 research articles pertaining to the research question. Among the 58 publications, there were 25 RCTs, 10 meta-analyses, and four systematic reviews, with the remaining publications including narrative reviews, post-hoc analyses, and comparative studies. The following section describes the major themes identified from these articles. Table 1 summarizes the comparison between ESAs and HIF-PHIs in terms of their efficacy and pharmacovigilance.

**Table 1:** Comparison of ESAs and HIF-PHIs: key differences in efficacy, safety, and administration ESAs, erythropoietin-stimulating agents; HIF-PHIs, hypoxia-inducible factor prolyl hydroxylase inhibitors; IV, intravenous

Feature	ESAs (epoetin alfa and darbepoetin alfa)	HIF-PHIs (roxadustat, daprodustat, and vadadustat)
Iron metabolism	Requires IV iron supplementation	Improves iron absorption and lowers hepcidin [11]
Administration	Injectable (subcutaneous/IV)	Oral tablets
Patient preference	Less preferred due to injections [12]	Higher satisfaction with oral administration [12]

Safety and cardiovascular risks

ESA-Associated Risks

High ESA doses were associated with increased cardiovascular risks, particularly in dialysis-dependent patients. A systematic review by Karimi et al. (2023) reported a 27% increased risk of cardiovascular mortality in hemodialysis patients receiving high-dose ESAs [10].

Cardiovascular Outcomes of HIF-PHIs

While roxadustat demonstrated a neutral cardiovascular risk profile, vadadustat was associated with a 1.17-fold increased risk of cardiovascular events according to the PRO2TECT trial [13]. This highlights the need for patient stratification when considering HIF-PHIs as an alternative to ESAs. Additionally, inflammation and oxidative stress modulation by HIF-PHIs may offer further advantages, but long-term cardiovascular safety remains uncertain [14].

Patient-Centered Outcomes

Higher hemoglobin levels were correlated with reduced fatigue and improved QoL [14]. The ASCEND-NHQ trial demonstrated that patients receiving daprodustat reported an improvement of 5.4 points in physical functioning scores (SF-36 vitality score) compared to placebo [15]. Similarly, molidustat-treated patients exhibited greater treatment satisfaction, with 70% preferring oral administration over injectable ESAs [12].

Innovations in dosing and administration

Fixed-dose ESA regimens reduced drug exposure while maintaining efficacy. A trial by Toto et al. (2020) found that a fixed-dose regimen of ESA (darbepoetin) had a similar transfusion requirement rate compared to titration-based dosing regimens with lower cumulative doses [16]. Oral HIF-PHIs, such as daprodustat, provide a convenient alternative to injectable ESAs, particularly in non-dialysis patients [15]. Additionally, intermittent dosing strategies (e.g., three times weekly) have shown comparable efficacy to the conventional IV epoetin alfa regimen with similar side effect percentages (75% and 78%, respectively) [17].

Alternative and adjunctive therapies

SGLT2 Inhibitors

Emerging evidence suggests that SGLT2 inhibitors may have erythropoietic benefits, leading to hemoglobin increases of 0.7 g/dL in CKD patients independent of EPO stimulation [18]. These agents have also been linked to improved cardiovascular outcomes (such as heart failure hospitalization) and slowed CKD progression, making them an attractive adjunct to anemia management [19].

Iron Supplementation

Intravenous (IV) iron formulations remain essential in anemia treatment. A double-blinded RCT has shown that ferric maltol increased hemoglobin levels in addition to all iron indices in stage III or IV CKD patients with iron deficiency anemia (IDA) compared to placebo [20]. Another open-label RCT compared two iron formulations (ferric citrate and ferrous sulfate) and showed the superiority of ferric citrate in increasing transferrin saturation and ferritin levels in moderate to severe CKD patients and IDA [21]. Additionally, ferric citrate has been linked to lower inflammation markers (CRP and IL-6) and reduced oxidative stress in CKD patients [22].

Role of ESAs in CKD-associated anemia in cancer

ESAs remain a key treatment modality for anemia in CKD patients with malignancies. A study by Deak et al. (2016) reported that while ESAs pose thromboembolic risks (HR=1.5), their positive effect on chemotherapy-induced anemia (CIA) has been shown, proving their role in managing patients with CIA [9]. As a result, personalized dosing strategies based on CKD severity and inflammation status have been proposed to minimize adverse effects while maintaining efficacy [7]. Table 2 briefly recapitulates the aforementioned points, outlining the main research findings concerning both conventional ESAs and alternative therapeutic interventions.

**Table 2:** Summary of findings ESAs, erythropoietin-stimulating agents; HIF-PHIs, hypoxia-inducible factor prolyl hydroxylase inhibitors; IV, intravenous

Key area	Summary of findings
ESA efficacy	ESAs effectively increase hemoglobin levels but at the cost of cardiovascular risks, particularly at high doses.
HIF-PHI efficacy	HIF-PHIs provide comparable hemoglobin increases with safety advantages, particularly regarding iron metabolism.
Safety concerns	Vadadustat has a higher cardiovascular risk, while roxadustat has a neutral safety profile.
Patient outcomes	HIF-PHIs improve quality of life, with SGLT2 inhibitors emerging as a promising adjunct.
Iron supplementation	IV iron therapy enhances anemia treatment and may reduce inflammation-related complications.

HIF-PHIs, therefore, represent a promising alternative to traditional ESAs in CKD-related anemia, with a potentially safer cardiovascular profile and additional benefits in iron metabolism. However, long-term safety concerns, particularly with vadadustat, highlight the need for patient-specific treatment strategies. Future research should prioritize long-term cardiovascular outcomes, comparative effectiveness studies, and cost-benefit analyses between ESAs, HIF-PHIs, and adjunctive therapies such as SGLT2 inhibitors and novel iron formulations.

Pathophysiology

Anemia of CKD is common and multifactorial in origin, with its primary causes including decreased renal production of EPO, altered iron metabolism, inflammatory responses, and factors affecting red blood cell survival [23].

EPO's decrease has recently been linked with the downregulation of hypoxia-inducible factor (HIF), which is a primary transcription factor that regulates gene expression of EPO [24].

EPO is produced by peritubular type 1 interstitial cells in the outer medulla and renal cortex and aids in the differentiation of erythroid cells in response to hypoxia. Without EPO, programmed apoptosis leads to the death of erythroid precursors. Simultaneously, pro-inflammatory cytokines released by damaged cells inhibit EPO production and reduce erythroid progenitor cells' ability to proliferate [23].

This bound iron is transferred to the liver and spleen and is either stored in ferritin or transported into the bone marrow for erythropoiesis. Moreover, iron is recycled by macrophages, which phagocytose senescent RBCs, which is another process dependent on EPO. This distinction may cause diagnostic challenges, as CKD-related anemia is typically normochromic, whereas IDA often presents as hypochromic and microcytic [24].

HIF is a transcription factor and plays a major role in cellular responses to hypoxia as it regulates EPO and iron-metabolism genes. When the levels are normal, it is hydroxylated and allows the von Hippel Landau protein to degrade it. In hypoxia, HIF is stabilized, which leads to increased EPO transcription, explaining how HIF indirectly decreases hepcidin levels through an increase in erythroferrone secretion by erythroblasts in CKD [24].

Optimizing ESA use: guidelines, risks, and future directions

Several studies done with regard to treatment of anemia in CKD have shown epoetin alfa and darbepoetin to be the most researched ESAs being put to use currently [22].

In the management of anemia in CKD and cancer-related conditions, the general consensus has shown that aiming for a higher hemoglobin target has been associated with increased adverse events. As such, use of ESAs in these conditions should be with a cautious approach. In effect, it is essential that more research is done to throw more light on the use of ESAs effectively in nephrology and oncology while mitigating the side effects from therapy, as there is limited data [9].

The severity of anemia in CKD often correlates with hemoglobin levels and the presence of comorbidities, such as diabetes or cardiovascular disease [8]. Notably, younger non-dialysis chronic kidney disease (ND-CKD) patients without diabetes demonstrated greater improvement in fatigue when treated to higher hemoglobin targets (11.7-13.5 g/dL) compared to current clinical practice levels (10-11.5 g/dL). This finding underscores the potential role of individualized treatment targets, particularly in patients with fewer complications. It also suggests that factors like age and comorbidity burden may influence the relationship between hemoglobin levels, fatigue, and overall health-related QoL.

Current guidelines for treating CKD-related anemia recommend specific hemoglobin targets and ESA-dosing strategies. Despite variations in pharmacokinetics and pharmacodynamics, such as differing half-lives among ESA types, no substantial evidence supports the clinical superiority of one type over another [24].

These findings emphasize the need for additional patient-centered studies to explore the effects of varying target hemoglobin levels on QoL. Furthermore, more observational studies and RCTs are required to comprehensively evaluate the efficacy, safety, and risks associated with different ESA types.

Over time, another category of drugs, HIF-PHIs, was introduced with the aim of providing better safety profiles and enhanced treatment of anemia in patients unable to tolerate ESAs. Although there has been general evidence that HIF-PHIs contribute to faster hemoglobin target, results of phase 3 trials done to support the improved safety profiles of HIF-PHIs with regard to cardiovascular complications have revealed a general non-inferiority to ESAs [2].

There has been an increasing significance of studies related to HIF-PHIs to improve their efficacy and utilization in anemia of CKD and cancer-related anemia. Hence, more evidence-based research needs to be carried out in the treatment of anemia with ESAs [7].

Mortality and Cardiovascular Risks Associated With ESA Therapy/Dosage-Related Mortality Risks

ESAs have been widely used for years for CKD-related anemia. They are effective in increasing hemoglobin level, improving health-related QoL, and decreasing the need for blood transfusions [8].

A comprehensive network meta-analysis evaluated the efficacy and safety of various ESAs, including epoetin alfa, epoetin beta, darbepoetin alfa, methoxy polyethylene glycol-epoetin beta, and biosimilar ESAs, compared to placebo or no treatment and found that ESAs effectively increased hemoglobin levels and reduced the need for red blood cell transfusions in individuals with CKD-related anemia [14]. However, the analysis did not find significant differences in efficacy among the different ESAs evaluated, adding to a potential limitation of the study.

They were noted to be associated with increased risk of hypertension, stroke, and vascular thrombosis. Also, higher hemoglobin targets achieved via ESA therapy were linked to an increased risk of mortality overall and serious cardiovascular events. The analysis also highlighted a trend where targeting higher hemoglobin levels with ESAs did not necessarily provide any additional benefits and was associated with greater risks instead. This suggests that a more conservative approach to hemoglobin targets may be safer for patients with CKD.

ESAs, especially in high doses and when hemoglobin and hematocrit levels were higher than recommended levels, led to adverse events. This effect was particularly discussed in the Normal Hematocrit Cardiac Trial (NHCT), where normal levels of hematocrit (42±3%) were compared to lower levels (30±3%), and showed increased mortality rates in the first two years and events such as vascular access thrombosis [2].

This effect is particularly significant in patients with ESRD, because they usually require higher doses of ESAs, which can cause more major adverse events without a significant increase in hemoglobin level [4]. This could also be explained by hypo-responsiveness caused by anemia of chronic inflammation. IL-6-induced hepcidin expression is a key mediator of such inflammation, which is targeted by ziltivekimab, a novel anti-IL-6 ligand antibody [20]. Hence, this led to the exploration of other alternatives and a growing need to evaluate newer therapies like HIF-PHIs to address limitations associated with ESA therapy while improving the safety and efficacy profile.

How Are HIF-PHIs a Safer Alternative?

HIF-PHIs are orally active first-in-class new generation drugs for renal anemia (Wang et al., 2020) [25]. HIF-PHIs have been reported to cause a reduction in serum hepcidin and ferritin as well as an increase in transferrin and total iron binding capacity (TIBC).

As mentioned in the Pathophysiology section, in patients with CKD, serum hepcidin is usually high secondary to reduced GFR and the presence of (subclinical) inflammation [25]. The reduction in hepcidin promotes the transport, mobilization, and utilization of iron. This occurs because a reduction in blood hepcidin level leads to increased absorption of iron from the intestinal tract and release of iron into the blood from hepatocytes and macrophages, resulting in higher iron levels in the blood.

Furthermore, HIF-PHIs can directly induce hepatic and renal EPO expression and increase the transcription of several iron transport and metabolism genes, finally promoting erythropoiesis by improving iron transport [26]. In comparison to ESAs, patients treated with HIF-PHIs have lower plasma EPO, reducing the risk of cardiovascular events and mortality [27]. So far, seven different PHIs, including roxadustat, vadadustat, daprodustat, molidustat, enarodustat, desidustat, and DS-1093a, are being investigated in more than 100 clinical trials. Of these, roxadustat was the first to be licensed to treat anemia in patients with DD-CKD (dialysis-dependent chronic kidney disease) and NDD-CKD (non-dialysis-dependent chronic kidney disease), and it is the most studied globally.

The potential advantages of HIF-PHIs over ESAs in the treatment of renal anemia include (i) raising hemoglobin without the risk of increasing blood pressure (BP); (ii) reducing the need for iron replacement therapy and blood transfusions [14]; (iii) effectiveness in patients resistant to ESAs due to microinflammation; and (iv) increased compliance as they are oral medication rather than injection [25].

A meta-analysis of 26 RCTs involving 2804 patients completed in 2020 concluded that HIF-PHIs showed a more favorable effect than placebo and were at least as efficacious as classic rhEPO in the short-term treatment of anemia in patients with CKD [25]. Some studies distinguished the effect of HIF-PHIs in comparison to ESAs on DD-CKD and NDD-CKD patients.

A meta-analysis by Zheng et al in 2023 analyzed 20 studies involving 14,737 participants with DD-CKD. This study showed that HIF-PHIs significantly increased iron, TIBC, and transferrin levels compared with ESAs. In contrast, hepcidin levels and the dosage of IV iron were significantly decreased in the HIF-PHIs group compared with the ESAs group [11]. Inflammation is one of the underlying causes of anemia in CKD and increases the dose requirements of rhEPO in ESRD patients. However, the dose requirements of HIF-PHIs such as roxadustat to maintain erythropoietic response were found to be unaffected by baseline CRP [11].

In 2023, Yang et al. compared the effects of HIF-PHIs, ESAs, and placebo on iron metabolism in renal anemia patients with NDD-CKD. The study showed that the ability of HIF-PHIs to correct anemia is not inferior to ESAs. Compared with placebo and ESAs, HIF-PHIs significantly decreased hepcidin, TSAT, and ferritin and increased transferrin and TIBC but did not alter serum iron [26]. Multiple trials involving HIF-PHIs showed non-inferiority in safety to ESAs, except for vadadustat in the non-dialysis population [28,29].

Emerging Alternatives: SGLT2 Inhibitors in CKD-Related Anemia

One of the selected review articles by Packer (2023) showed that, according to large-scale clinical trials, sodium-glucose cotransporter-2 (SGLT-2) inhibitors have the potential to effectively increase hemoglobin and hematocrit in patients with CKD-associated anemia. SGLT-2 inhibitors are a group of medications originally used to treat type 2 diabetes mellitus and are also widely used in CKD and chronic heart failure due to their cardiovascular and nephroprotective benefits [19]. The article also compares this class of medications with HIF-PHIs and explains that both groups have similar biochemical pathways for regulating and promoting red blood cell production.

However, HIF-PHIs have additional, undesirable effects on alternate biochemical pathways that activate intravascular inflammatory pathways, which may aggravate existing cardiovascular or kidney injury [30]. The study suggests that this effect is not seen with SGLT-2 inhibitors, making them a safer alternative to HIF-PHIs from a renal and cardiovascular perspective. Therefore, as a potential therapeutic option for renal anemia, further research is needed to explore the use of SGLT-2 inhibitors in clinical settings for this indication.

ESAs in the oncology setting

The literature search found four articles that explored the use of ESAs in the context of cancer-related anemia. Multiple potential causes of anemia in cancer include cancer-related inflammation, iatrogenic effects from chemotherapy agents, and poor nutritional intake or absorption. Selected articles [9,7] indicated that, although ESAs reduce the requirement for blood transfusions and improve functional outcomes in cancer-related anemia, they also increase the risk of venous thromboembolism (VTE) in patients with a pre-existing risk and accelerate rates of adverse cardiovascular events and mortality at higher ESA doses. Therefore, the 2018 ESMO Clinical Practice Guidelines on ESA treatment for cancer-related anemia restrict the target hemoglobin level to below 12 g/dL and recommend avoiding this treatment in patients with known thromboembolic disorders or uncontrolled hypertension [7].

Future research should focus on revising the recommended indications for ESA use and exploring potentially safer therapeutic alternatives that promote erythropoiesis in cancer-related anemia, while reducing the risk of cardiovascular events and avoiding influence on existing cancer progression. This could allow the benefits of cancer-related anemia treatment to reach a broader range of patients.

Overall effectiveness and risks of ESA

In 1989, ESAs were approved by the U.S. FDA for the treatment of anemia. Subsequent studies continued to show the benefits of ESAs, including improved QoL, neurocognitive function, cardiac function, and regression of left ventricular hypertrophy [7]. In 2006, they became one of the mainstays of treatment for anemia of CKD, along with iron supplementation. The use of rhEPO improved anemia symptoms, such as dyspnea, palpitations, and fatigue, thereby reducing the need for blood transfusions in transfusion-dependent patients with CKD [15,16].

Although ESAs have proven beneficial for treating anemia in CKD and CIA, they have limitations. The two main causes of ESA hypo-responsiveness in patients with anemia in CKD on dialysis are iron deficiency and systemic inflammation [11]. Various RCTs and meta-analyses found that using ESAs to achieve higher hemoglobin targets (above 12 g/dL) resulted in increased cardiovascular and cerebrovascular side effects, tumor progression, recurrence, and mortality [7].

The mechanism behind ESA-related cardiovascular side effects remains unknown. Large ESA doses and rapid hemoglobin increases have been suggested as contributing factors to these risks [16].

In patients with a history of seizures, darbepoetin alfa should be used cautiously, especially during the first few months, as it may cause seizures and has been associated with hypertension and progression of certain malignancies [3].

ESAs also require strict cold-chain storage to preserve stability and prevent immunogenicity, which may limit access in some regions. Given their cost, their availability is limited in many countries. Epoetin medications are injectable, making them less desirable for patients [2].

Benefits and risks of HIF-PHIs

HIF stabilizers have been shown to reduce the need for blood transfusions in patients with chronic kidney disease. They have also increased the number of patients who achieved improved hemoglobin levels. However, their effects on life expectancy and cardiac pathology in patients with CKD remain uncertain [14].

HIF-PHI drugs have been shown to improve iron availability, allowing for lower iron supplementation. Improvements in inflammation and immune function have also been observed. In inflamed patients, these medications effectively decrease hepcidin directly or indirectly. HIF-PHIs have additionally been shown to lower serum cholesterol by inhibiting cholesterol synthesis and enhancing intestinal excretion [2]. Adverse events were infrequently reported; for example, hyperkalemia was mentioned but not clearly defined [14].

ESAs and HIF-PHIs offer distinct advantages and limitations in renal anemia. Epoetin alfa and darbepoetin alfa have been well-established since their FDA approval in 1989, significantly reducing transfusion dependency and improving anemia-related symptoms, cardiac function, and QoL [4]. However, ESA risks, including cardiovascular events, cerebrovascular complications, VTE, hypertension, and increased mortality at high hemoglobin targets, limit their safety profile [3,8]. HIF-PHIs, in contrast, provide an oral alternative that raises hemoglobin levels while improving iron bioavailability and reducing inflammation, potentially lowering the need for iron supplementation [2]. Despite these benefits, uncertainties remain regarding their long-term effects on mortality and cardiovascular outcomes. HIF-PHIs also affect cholesterol metabolism, but their adverse effects, such as hyperkalemia, require further evaluation [2,14]. Ultimately, choosing between ESAs and HIF-PHIs requires careful consideration of efficacy and safety, with additional research needed to determine long-term outcomes for both drug classes in CKD anemia management.

Regarding the choice of either class, limited data exist on the long-term safety and efficacy of HIF-PHIs, including their potential to cause hyperkalemia. This area requires further exploration and has not been widely addressed in clinical trials. Overall, the long-term impact of ESAs and HIF-PHIs on cardiovascular health and overall survival remains unclear, and further research is essential to establish evidence-based conclusions. It is also necessary to identify patient populations who would benefit most from one treatment over the other. Given current data gaps, ongoing trials and studies will be crucial in guiding future treatment decisions.

Alternatives and balancing risks and benefits in oncology

In oncology, determining whether to use ESAs or HIF-PHIs for anemia remains challenging due to differences in efficacy, safety, and clinical context, particularly because their efficacy profiles, risk factors, and practical aspects in clinical practice differ. Epoetin alfa and darbepoetin alfa are mainly used to treat anemia in cancer patients on chemotherapy. They effectively improve anemia-related symptoms, reduce transfusion dependence, and enhance QoL [7]. However, as discussed earlier, they carry risks and may increase mortality rates, especially when targeting elevated hemoglobin levels. ESAs may also exacerbate tumor progression in certain cancers, creating additional challenges in patients undergoing chemotherapy. HIF-PHIs, if thoroughly studied for long-term outcomes and risks, could potentially mitigate some safety concerns [2]. Throughout this review, data on the long-term effects of HIF-PHIs on mortality and cardiovascular outcomes remain inconclusive, and their potential for adverse effects, such as hyperkalemia, presents a gap that requires careful analysis and further study [14]. HIF-PHIs offer the advantage of oral administration, which is more convenient than injectable ESAs.

In oncology, while both drug groups show promise, significant gaps remain in understanding their long-term efficacy and potential interactions with cancer therapies [30]. Despite progress in managing anemia in cancer patients, the long-term outcomes of both agents, particularly in combination with chemotherapy, and potential interactions are still under investigation, and many questions remain unanswered. Balancing the risks and benefits of ESAs and HIF-PHIs is therefore ideal. This review highlights the need for further research to better define the appropriate use of these agents in cancer-related anemia. Critically comparing the strengths and limitations of ESAs and HIF-PHIs is essential to establishing a comprehensive understanding of their clinical roles and guiding future treatment strategies effectively.

Strengths and limitations

A limitation of this review is its qualitative nature, with articles selected based on a specific set of keywords deemed relevant to the utilization of ESAs and the use of only PUBMED for the article search. Another limitation is the time frame within which the data were collected and analyzed. Relying primarily on data from the last 5 years may result in missing potentially important earlier articles on ESAs.

However, this also strengthens the review, as recent guidelines on ESA use in anemia of CKD and cancer-related conditions were comprehensively reviewed, allowing for relevant recommendations to improve ESA efficacy and safety according to current standards. The ever-changing nature of medicine makes the time frame applied in this review important for aligning our recommendations with future research on the current use of ESAs in CKD and cancer-related anemia.

## Conclusions

This narrative review highlights that the use of ESAs in CKD-related anemia improves QoL and reduces transfusion needs but is consistently associated with increased cardiovascular risk, higher mortality, and, in the oncology setting, tumor progression at higher doses. Evidence from recent studies also suggests that targeting higher hemoglobin thresholds provides no additional benefit and may worsen outcomes, underscoring the need for cautious use and individualized dosing. These findings align with current KDIGO recommendations, which advocate conservative hemoglobin targets and emphasize balancing benefits with risks when prescribing ESAs.

Emerging alternatives, particularly HIF-PHIs, demonstrate comparable efficacy with added advantages in iron metabolism, oral administration, and patient adherence, although long-term safety data remain limited. Adjunctive therapies such as SGLT-2 inhibitors and novel iron formulations also hold promise as complementary options, offering a broader therapeutic toolkit for anemia management in CKD and cancer populations. Overall, this review emphasizes the importance of additional RCTs and long-term observational studies to evaluate the comparative safety, efficacy, and patient-centered outcomes of ESAs, HIF-PHIs, and adjunctive therapies. By addressing these gaps, future research can better inform individualized, safe, and effective treatment strategies for patients with anemia in CKD and cancer.
